# *Toxoplasma gondii* exposure may modulate the influence of *TLR2* genetic variation on bipolar disorder: a gene–environment interaction study

**DOI:** 10.1186/s40345-016-0052-6

**Published:** 2016-05-20

**Authors:** José Oliveira, Rémi Kazma, Edith Le Floch, Meriem Bennabi, Nora Hamdani, Djaouida Bengoufa, Mehdi Dahoun, Céline Manier, Frank Bellivier, Rajagopal Krishnamoorthy, Jean-François Deleuze, Robert Yolken, Marion Leboyer, Ryad Tamouza

**Affiliations:** INSERM, U1160, Hôpital Saint Louis, Paris, France; Fondation FondaMental, Créteil, France; Centre National de Génotypage, CEA, Evry, France; Faculté de Médecine, Université Paris-Est, Créteil, France; AP-HP, DHU PePSY, Pôle de Psychiatrie, Hôpitaux Universitaires Henri Mondor, Créteil, France; INSERM, U955, Psychiatrie Translationnelle, Créteil, France; Laboratoire Jean Dausset and LabEx Transplantex, Hôpital Saint Louis, Paris, France; Sorbonne Paris-Cité, Université Paris Diderot, Paris, France; INSERM UMR-S1144-VariaPsy, Hôpital Fernand Widal, Paris, France; Stanley Laboratory of Developmental Neurovirology, Johns Hopkins University Medical Center, Baltimore, USA; Stanley Research Program, Sheppard Pratt, Baltimore, MD USA

**Keywords:** Bipolar disorder, Innate immunity, Pathogen recognition receptor, Genetic diversity, Infection, Gene–environment interaction

## Abstract

**Background:**

Genetic vulnerability to environmental stressors is yet to be clarified in bipolar disorder (BD), a complex multisystem disorder in which immune dysfunction and infectious insults seem to play a major role in the pathophysiology. Association between pattern-recognition receptor coding genes and BD had been previously reported. However, potential interactions with history of pathogen exposure are yet to be explored.

**Methods:**

138 BD patients and 167 healthy controls were tested for serostatus of *Toxoplasma gondii*, CMV, HSV-1 and HSV-2 and genotyped for *TLR2* (*rs*4696480 and *rs*3804099), *TLR4* (*rs*1927914 and *rs*11536891) and *NOD2* (*rs*2066842) polymorphisms (SNPs). Both the pathogen-specific seroprevalence and the *TLR/NOD2* genetic profiles were compared between patients and controls followed by modelling of interactions between these genes and environmental infectious factors in a regression analysis.

**Results:**

First, here again we observed an association between BD and *Toxoplasma gondii* (*p* = 0.045; OR = 1.77; 95 % CI 1.01–3.10) extending the previously published data on a cohort of a relatively small number of patients (also included in the present sample). Second, we found a trend for an interaction between the *TLR2rs*3804099 SNP and *Toxoplasma gondii* seropositivity in conferring BD risk (*p* = 0.017, uncorrected).

**Conclusions:**

Pathogen exposure may modulate the influence of the immunogenetic background on BD. A much larger sample size and information on period of pathogen exposure are needed in future gene–environment interaction studies.

## Background

Bipolar disorder (BD) is a heritable chronic psychiatric illness standing among the leading causes of disability worldwide (Collins et al. 2011; Gore et al. 2011; Whiteford et al. 2013). The aetiology of BD is multifactorial and results from complex interactions between genetic determinants and a number of known and yet to be uncovered environmental factors (Kerner 2014). The index bipolar diagnosis is often delayed by many years as longitudinal natural history is one among the components of diagnosis (Leboyer and Kupfer 2010). Currently, objective biomarkers that could lead to early and specific diagnosis of BD are lacking and identification of such markers along with objectively assessed environmental factors could assist in tailoring reasoned preventive and therapeutic approaches for BD.

Studies on gene–environment interactions in psychiatric disorders essentially focus on early-life psychosocial stressors as the environmental risk factor and to a limited extent on the infectious history of the mother (during pregnancy) or the foetus/neonate (Uher 2014). For example, in BD, an interaction between *BDNF* gene polymorphisms and stressful life events seems to confer relatively higher risk for depressive episodes (Hosang et al. 2010). Similarly, maternal herpes simplex virus 2 (HSV-2) and cytomegalovirus (CMV) infectious history appears to interact, respectively, with *GRIN2B* and *CTNNA3* gene polymorphisms to raise the risk of schizophrenia (SZ) (Børglum et al. 2014; Demontis et al. 2011). While the assessment of early-life stress may be subject to recall bias, history of infections is relatively amenable to objective evaluation. Indeed, in recent studies, infectious agents emerged as a group of well-defined environmental risk factors in psychiatric disorders and in particular in BD (Canetta et al. 2014; Dickerson et al. 2004; Hamdani et al. 2013; Parboosing et al. 2013). These studies not only stress the importance of studying infectious events but also, by decreasing the sample heterogeneity, allow to capture the environmental influence on genetic liability (Kazma et al. 2011). Genetic susceptibility to infections is mediated by the inter-individual variability of immune responses, the first line defence being the innate immune system (Kotb et al. 2002; Pandey et al. 2014).

In this regard, genetic polymorphism of the pattern-recognition receptors (PRRs) is of interest as PRRs recognize the pathogen-associated molecular patterns (PAMPs) (bacterial, viral, fungal or parasite-derived) as well as host-derived danger signals to provide optimal protection (Akira et al. 2006). We recently provided evidence for associations between BD and genetic variants of *TLR2*, *TLR4* and *NOD2* genes, all encoding for pivotal PRRs (Oliveira et al. 2014a, b, c). The membrane-bound TLR2 and TLR4 and the cytoplasmic NOD2 receptors are widely expressed on immune cells, intestinal epithelial cells and more importantly in microglia that actively participate in the central nervous system (CNS) homeostasis and immune surveillance (Akira et al. 2006; Berrebi et al. 2003; Hanke and Kielian 2011; Lala et al. 2003; Olson and Miller 2004; Rivest 2009; Sterka and Marriott 2006).

Although genetic associations between *TLR2*, *TLR4* and *NOD2* polymorphisms with susceptibility to infections on the one hand (Schröder and Schumann 2005; Tekin et al. 2012; van Well et al. 2013), and BD (as discussed above) on the other have been independently reported in the literature, the influence of interaction between a particular infection and variability of the innate immune system on BD phenotype remains to be tested.

In order to explore possible gene–environment interactions, the present study evaluates the effect of interactions between serologically documented exposure to *Toxoplasma gondii*, CMV, HSV-1 or HSV-2 and polymorphisms of *TLR2*, *TLR4* and *NOD2* genes in a sample of patients with BD.

## Methods

### Subjects

This study included 138 cases (in- and out-patients) meeting the DSM-IV criteria (American Psychiatric Association 1994) for BD [type I, II or not otherwise specified (NOS)], from two French university-affiliated psychiatry departments (Mondor Hospital, Créteil, University Paris-Est and Fernand Widal Hospital, Paris, University Paris Diderot).

Patients were interviewed with the French version of the “Diagnostic Interview for Genetic Studies” (DIGS) for the assessment of lifetime clinical characteristics of BD as well as for demographic characteristics (i.e. number of years of education, working status, place of birth) (Nurnberger et al. 1994). Ongoing treatments as well as hospitalization status were recorded. Manic and depressive symptoms were assessed using, respectively, the Young Mania Rating Scale (Young et al. 1978) and the Montgomery–Åsberg Depression Rating Scale (Montgomery and Asberg 1979). In addition, 167 healthy controls were enrolled through a clinical investigation centre (Centre for Biological Resources, Mondor Hospital, Créteil, France). Only subjects without any personal or family history of psychotic disorders, affective disorders, addictive or suicidal behaviour, or auto-immune diseases were included in the present study. Patients and controls were submitted to serological screening and were negative for HIV-1/-2, Hepatitis A, B and C and had no known recent inflammatory, auto-immune or infectious event or a neurological disease. Knowing that *Toxoplasma gondii* infection incidence highly varies according to geographical location and assuming that potential early-life influences of *Toxoplasma gondii* may thus vary according to place of birth, a subsample including only individuals born in Metropolitan France was selected to study the association between this parasite and BD. Indeed, to refine the genetic association analysis, a homogeneous subgroup of 76 cases and 67 controls, with French ancestry (at least 3 grandparents born in mainland France), was selected. Written informed consent was obtained from all participating subjects and the institutional ethical committee approved the research protocol.

### Serological analysis

Solid-phase enzyme microplate immunoassay methods were used to measure the IgG and IgM class antibodies to *Toxoplasma gondii*, CMV, HSV-1 and HSV-2 in blood samples using previously described methods (Yolken et al. 2001). Assay reagents were obtained from IBL America (Minneapolis, Minnesota, USA). The results were expressed as a ratio between the reactivity of the sample and a standard sample run on each microplate. Seropositivity was defined as a *T. gondii* IgG ratio ≥0.8, equivalent to ≥10 international units. All antibody measurements were carried out at the Stanley Laboratory of Developmental Neurovirology, Baltimore, MD, USA. All samples were coded to ensure anonymity and the laboratory did not have access to diagnostic or clinical information.

### *TLR2*, *TLR4* and *NOD2* genotyping

Genomic DNA was extracted from EDTA-treated peripheral blood sample using a standard procedure. Selection of analysed single-nucleotide polymorphisms (SNPs) of *TLR2* (*rs*4696480 and *rs*3804099), *TLR4* (*rs*1927914 and *rs*11536891) and *NOD2* (*rs*2066842) were based on previous positive genetic association findings with BD subsets (Oliveira et al. 2014a, b, c). All SNPs were analysed by a pre-developed TaqMan^®^ 5′-nuclease assay kits (Applied Biosystems^®^, Foster City, CA, USA) using allele-specific fluorogenic oligonucleotide probes as described earlier (Oliveira et al. 2014a, b, c).

### Statistical analysis

The comparison of demographic/clinical characteristics and *Toxoplasma gondii*, CMV, HSV-1 and HSV-2 serological status between cases and controls was performed using Chi-square tests for categorical variables and Mann–Whitney *U* tests for continuous variables. To correct for multiple testing (four serological markers), we used the Bonferroni approach to determine the significance threshold (0.05/4 = 0.0125). According to these results, a multiple logistic regression analysis was further performed to test the association between *Toxoplasma gondii* seropositivity and BD diagnosis adjusting for age at inclusion and level of education (higher education vs other). This logistic regression analysis was similarly performed in the subsample of individuals born in France.

Allelic frequency comparisons between cases and controls and testing for deviation from Hardy–Weinberg proportions in controls were performed using the Chi-square tests. Fisher’s exact test was used wherever appropriate.

To test for the potential gene–environment interaction between the studied SNPs of *TLR2*, *TLR4* and *NOD2* and *Toxoplasma gondii* serostatus, we modelled a multiple logistic regression incorporating an interaction term, also adjusting for age at inclusion and level of education. The number of minor alleles was included in the model as a continuous variable, in our additive genetic model. To correct for multiple testing (five SNPs), we used the Bonferroni approach to determine the significance threshold (0.05/5 = 0.01). All associations were first tested for the whole sample and in a second step using only the subsample of individuals of French ancestry (as described above). All statistical analyses were performed using the SPSS v20.0.0 and the WINPEPI v11.43 software packages (Abramson 2011).

## Results

Demographic/clinical characteristics and allele distributions in cases and controls are given, respectively, in Tables 1 and 3. The distribution of all the studied SNPs complied with the expected Hardy–Weinberg proportions.

**Table 1:** Clinical and demographic characteristics of bipolar and control subjects

	BD (*n* = 138)	HC (*n* = 167)	*p* value
Mean ± SD			
Age at inclusion (years)	43.6 ± 13.50	39.5 ± 13.77	0.009
Age at onset (years) (*n* = 135)^a^	26.3 ± 10.51	–	–
*n* (%)			
Gender (female)	71 (51.4)	90 (53.9)	0.671
Diagnoses (DSM-VI)			
BD-I	98 (71.0)	–	–
BD-II	37 (26.8)	–	–
BD-NOS	3 (2.2)	–	–
Place of birth (Metropolitan France)	113 (81.9)	137 (82.0)	0.973
Education level (higher education)	78 (56.5)	72 (43.1)	0.020
Working status (currently active)	72 (52.2)	93 (55.7)	0.540
Serum anti-pathogen IgG (positive)			
Toxoplasma gondii	103 (74.6)	97 (58.1)	0.002
CMV	84 (60.9)	107 (64.1)	0.565
HSV-1	34 (24.6)	47 (28.1)	0.490
HSV-2	87 (63.0)	119 (71.3)	0.127

*BD* bipolar disorder; *HC* healthy controls; *NOS* not otherwise specified; *SD* standard deviation; *CMV* cytomegalovirus; *HSV*-*1* herpes simplex virus 1; *HSV*-*2* herpes simplex virus 2; *NS* non-significant

^a^Data were missing for three patients

We found a statistically significant association between BD and *Toxoplasma gondii* IgG seropositivity (univariate *p* value = 0.002) which was additionally confirmed by a multiple logistic regression analysis adjusting for age at inclusion and level of education (*p* value = 0.049; OR = 1.77; 95 % CI 1.00–3.11) (Table 2). We also observed this association in the subgroup of patients born in France, similarly adjusting for age at inclusion and level of education (*p* value = 0.009; OR = 2.34; 95 % CI 1.23–4.43) (Table 2). This result confirms and extends the previously published data on a smaller set of French patients belonging to the same cohort and included in the present study (101 BD patients and 95 healthy controls in common) (Hamdani et al. 2013).

**Table 2:** Association between *Toxoplasma gondii* and bipolar disorder adjusted for age and level of education in the whole sample

	Entire sample					Individuals born in France				
	*β*	OR	CI 95 %	df	*p* value	*β*	OR	CI 95 %	df	*p* value
Age	0.016	1.016	0.996–1.036	1	0.110	0.009	1.009	0.988–1.031	1	0.403
Level of education	0.629	1.876	1.172–3.003	1	0.009	0.656	1.928	1.144–3.248	1	0.014
*Toxoplasma gondii*	0.568	1.765	1.002–3.108	1	0.049	0.850	2.339	1.234–4.433	1	0.009

Level education = higher education vs other level of education

*OR* odds ratio; *CI* confidence interval; *df* degrees of freedom

None of the five SNPs studied in *TLR2*, *TLR4* and *NOD2* were significantly associated with BD, using an allelic or genotype model after accounting for multiple testing and remained non-significant for the French ancestry subset (data not shown).

A trend for an interaction between *TLR2rs*3804099 and *Toxoplasma gondii* influencing the risk for BD was noted, although no longer significant after correction for multiple testing (Table 3). This SNP has an odds ratio below one in the *Toxoplasma gondii* seropositive group and the opposite in the seronegative group as assessed by separate logistic regression analyses with an additive model (Table 4).

**Table 3:** *TLR2*, *TLR4* and *NOD2* polymorphisms in bipolar patients and healthy controls: genotype distribution

Polymorphism		MAF		*p* value	HWE *p* value
		BD (*n* = 138)	Controls (*n* = 167)		
*TLR2 rs*4696480	T > A	0.46	0.51	0.178	0.929
*TLR2 rs*3804099	T > C	0.46	0.44	0.610	0.804
*TLR4 rs*1927914	A > G	0.41	0.39	0.601	0.671
*TLR4 rs*11536891	T > C	0.17	0.14	0.265	0.588
*NOD2 rs*2066842	C > T	0.25	0.25	0.898	0.656

*TLR2* Toll-like receptor 2; *TLR4* Toll-like receptor 4; *NOD2* nucleotide-binding oligomerization domain containing 2; *BD* bipolar disorder; *NS* non-significant; *MAF* minor allele frequency; *HWE* Hardy–Weinberg equilibrium in controls

**Table 4:** SNP x *Toxoplasma gondii* interaction

Polymorphism		TG positive		TG negative		*p* value (GxE)	OR TG (+) (CI 95 %)	OR TG (−) (CI 95 %)
		MAF BD	MAF HC	MAF BD	MAF HC			
*TLR2* *rs*4696480	T/A	0.47	0.53	0.44	0.49	0.923	0.760 (0.51–1.14)	0.805 (0.44–1.47)
*TLR2 rs*3804099	T/C	0.42	0.45	0.59	0.43	0.021	0.894 (0.62–1.30)	2.289 (1.15–4.58)
*TLR4 rs*1927914	A/G	0.40	0.37	0.46	0.42	0.992	1.102 (0.76–1.60)	1.154 (0.65–2.05)
*TLR4 rs*11536891	T/C	0.17	0.11	0.17	0.18	0.341	1.546 (0.91–2.64)	0.944 (0.42–2.14)
*NOD2 rs*2066842	C/T	0.25	0.28	0.24	0.20	0.408	0.875 (0.56–1.37)	1.300 (0.64–2.63)

*SNP* single-nucleotide polymorphism; *TG* *Toxoplasma gondii*; *MAF* minor allele frequency; *BD* bipolar disorder; *HC* healthy controls; *GxE* gene–environment interaction; *OR* odds ratio; *CI* confidence interval

## Discussion

Inherited immune vulnerability to environmental risk factors may explain the association between BD and exposure to a variety of infections early in life, during a critical neurodevelopmental window (Canetta et al. 2014; Foldager et al. 2014; Graham et al. 2014; Hamdani et al. 2013; Mazaheri-Tehrani et al. 2014; Parboosing et al. 2013). Herein, we explored the interaction between infectious stigma and genetic variants of the innate arm of the immune response in a sample of adult BD patients.

First, we confirmed the influence of Toxoplasma infection in BD by analysing a much larger sample than in a previous study (Table 1) and also by stringently selecting those born in France for sample homogeneity (Hamdani et al. 2013). Second, we did not find a genetic association between individual *TLR2*, *TLR4* and *NOD2* alleles and BD, as compared to our previous positive findings of association in a distinct study cohort (Oliveira et al. 2014a, b, c). Such discrepancy could possibly be related to differences in the study design. The present study involves a relatively smaller sample size, specifically when considering only individuals of French ancestry, 76 cases and 67 controls. Such differing study setting may not necessarily have the power to detect the previously observed genetic associations, involving approximately 550 and 200 cases and controls, respectively, and stringently selected for their genetic homogeneity (at least three generations from mainland France) (Oliveira et al. 2014a, b, c). However, it must be noted that the referred SNPs were not associated with BD in the large GWAS by the Psychiatric Genomics Consortium (7481 patients and 9250 controls) (Sklar et al. 2011). Third, in the gene–environment interaction analysis, the *TLR2 rs*3804099/Toxoplasma interaction shows a trend in that the *TLR2* genetic variation may modulate the relationship between *Toxoplasma gondii* exposure and BD.

Indeed, TLR2 molecules have been implicated: (i) in the perpetuation of inflammatory responses in the CNS after pro-inflammatory stimulation of astrocytes (Henn et al. 2011); (ii) in triggering neuro-inflammation in the presence of endogenous molecules such as α-synuclein and amyloid β-peptide, respectively, in Parkinson and Alzheimer disorders (Kim et al. 2013; Liu et al. 2012); and (iii) in sustaining neuro-inflammation due to HSV-1 infection (Aravalli et al. 2005). Besides these observations, association of *TLR2* genetic diversity either with Alzheimer’s disease (Yu et al. 2011) or with cognitive function in SZ (Kang et al. 2013) suggests that inter-individual liability to modulate neuro-inflammation may be genetically driven. Yet direct evidence for the involvement of *TLR2* in susceptibility to *Toxoplasmagondii* and neuro-inflammation is lacking. Nevertheless, a role for *TLR2* is highly probable since higher loads of *Toxoplasma gondii* in brain tissue were observed in *TLR2*-deficient mice following infection with *Toxoplasma gondii* cysts (Mun et al. 2003).

We have recently described a combined effect of both *TLR2 rs*3804099 TT genotype and reported childhood sexual abuse on the age at onset of BD, a proxy of disease severity (Oliveira et al. 2015). Additionally, in a murine model, prenatal immune priming (through the TLR3 pathway) combined to peripubertal stress were shown to synergistically induce behavioural changes, unbalanced neurotransmitter levels, enhanced expression of markers of inflammation and microglia activation in stress-sensitive brain areas of mice (Giovanoli et al. 2013). We may thus hypothesize that aberrant encounters viz infectious insults and/or psychosocial stress early in life may affect neurodevelopmental processes in genetically vulnerable individuals through a common mechanism involving inherited defective innate immune responses (Dickerson et al. 2014; Foldager et al. 2014).

In our study, it must be noted that the *TLR2rs*3804099/*Toxoplasma gondii* interaction term in the regression analysis was not statistically significant after correction for multiple comparisons. However, the trend found for the *Toxoplasma gondii* x *TLR2* interaction and the observation that the *TLR2 rs*3804099 demonstrated an OR of 0.894 in the seropositive group and of 2.289 in the seronegative one emphasizes the pertinence of considering environmental risk factors when evaluating the genetic risk of BD and warrant replication using a much larger cohort. Another limitation of the present study is the absence of information regarding the ‘time window” of exposure to *Toxoplasma gondii* as we only have data on IgG antibodies.

## Conclusions

We extend previously published data confirming the association between *Toxoplasma gondii* and BD on a much larger sample size and suggest that history of Toxoplasma exposure may modulate the influence of *TLR2* polymorphism on BD. A much larger sample size and better characterization of environmental risk exposure containing information on type, intensity and period of pathogen exposure are needed in future studies. If infections were ever to play a significant role in BD pathogenesis in interaction with host genetic liability, one would expect that appropriate and timely interventions on the environmental insults must be able to prevent or reduce the pathogenic load of BD highlighting the importance of gene-environment studies.
